# Anthropogenic Microparticles in Sea-Surface Microlayer in Osaka Bay, Japan

**DOI:** 10.3390/jox13040044

**Published:** 2023-11-07

**Authors:** Mi Zhou, Hirofumi Yanai, Chee Kong Yap, Christina Emmanouil, Hideo Okamura

**Affiliations:** 1 Graduate School of Maritime Sciences, Kobe University, Fukaeminami-machi, Higashinada-ku, Kobe 658-0022, Japan; zhoumi77@163.com (M.Z.); yahirofumi1996@yahoo.co.jp (H.Y.); yapchee@upm.edu.my (C.K.Y.); 2 Department of Biology, Faculty of Science, Universiti Putra Malaysia, UPM Serdang, Serdang 43400, Selangor, Malaysia; 3 School of Spatial Planning and Development, Aristotle University of Thessaloniki, 54124 Thessaloniki, Greece; chemmanouil@plandevel.auth.gr; 4 Research Center for Inland Seas, Kobe University, Fukaeminami-machi, Higashinada-ku, Kobe 658-0022, Japan

**Keywords:** microparticles (MPs), seawater surface microlayer, PMMA, antifouling paint particles (APPs), Osaka Bay

## Abstract

The abundance, distribution, and composition of microparticles (MPs) in the sea-surface microlayer (S-SML, less than 100 μm of sea surface in this experiment) and in bulk water (1 m under the sea surface) were investigated to evaluate the pollution level of MPs in Osaka Bay in Japan. Both seawater fractions were collected at eight sites including ship navigation routes, the coastal area, and the center of Osaka Bay for 2021–2023. MPs were filtered for four size ranges (10–53, 53–125, 125–500, and >500 μm) and then digested with H_2_O_2_. MPs’ abundance was microscopically assessed; and polymer types of MPs were identified by a Fourier transform infrared spectrometer (FTIR). For the 22 collections performed along eight sites, the average MPs’ abundance was 903 ± 921 items/kg for S-SML, while for the 25 collections performed along the same sites, the average MPs’ abundance was 55.9 ± 40.4 items/kg for bulk water, respectively. MPs in both S-SML and bulk water exhibited their highest abundance along the navigation routes. The smallest MPs (10–53 μm) accounted for 81.2% and for 62.2% of all MPs in S-SML and in bulk water among all sites, respectively. Polymethyl methacrylate (PMMA) was the major type of MPs identified while minor ones were polyethylene, polyesters, polystyrene, polypropylene, polyvinyl chloride, polyamide, etc. PMMA comprised 95.1% of total MPs in S-SML and 45.6% of total MPs in bulk water. In addition, PMMA accounted for 96.6% in S-SML and 49.5% in bulk water for the smallest MP category (10–53 μm). It can be assumed that the MP sources were marine paints—primarily APPs (antifouling paint particles)—as well as land coatings. Sea pollution due to microparticles from ship vessels should be given proper attention.

## 1. Introduction

The presence of microplastics (plastic particles less than 5 mm) is a ubiquitous problem in terrestrial and aquatic ecosystems. Once introduced into an aquatic environment by wind transport, stormwater runoff, drainage, and sewage system, plastic debris can be transported offshore and enter oceanic gyres by surface currents and winds, which leads to 80% of terrigenous plastic waste accumulating in a marine environment [1,2]. For instance, at least 79 thousand tons of ocean plastics are floating inside an area of 1.6 million km^2^ at the Great Pacific Garbage Patch (GPGP), where microplastics account for 8% of the total mass [3]. Besides the residual of primary microplastics with microscopic sizes such as resin pellets and microbeads used in cosmetics and air-blasting media [4,5], secondary microplastics are continuously generated from the breakdown of larger plastic debris due to UV irradiation, mechanical forces and biodegradation by bacteria, fungi, and microalgae, and they also end up in marine ecosystems [6,7]. Compared to macroplastics, microplastics can exudate more toxic additives due to their large specific surface and their full exposure to seawater, and they can transmit hazardous chemicals to biota [8]. Additionally, microplastics are prone to adsorb persistent organic pollutants (POPs) such as PCBs and PBDEs from surrounding water medium [9]. Once these microplastics are ingested by all kinds of aquatic fauna including invertebrates, planktonic organisms, seabirds, crustaceans, fish, aquatic animal and marine mammals, they may bioaccumulate and biomagnify via the food web [10,11]. Microplastics have been discovered in a diversity of seafood items and even in human feces and blood clots [12,13]; as such, it can be assumed that humans are exposed to microplastics through their diet. This exposure may compromise fecundity and other somatic processes and potentially threaten human health [14].

It is assumed that 20% of marine-based plastic debris mainly stems from fishing gears, aquaculture, marine structures, and vessels [15]. Furthermore, antifouling paint particles (APPs) and other marine paint debris [15], can flake off from hulls during processes of shipbuilding, boat cleaning, and vessel navigation and then enter the local aquatic environment. This source has been incidentally or deliberately excluded from the characterization of microplastics due to the omission of paint in marine litter guidelines [16]. Nevertheless, some investigations have reported evidence of the occurrence of paint particles in seawater; paint microparticles in surface seawater accounted for 33% of all microplastics in the South China Sea [17] and 15.21% of all microplastics in the Jiangsu coastal area in China [18], respectively. In the sea-surface microlayer of the southern coast of Korea, floating alkyd resins (171 items/L) were predominant (81% of all microsized polymer particles) [11]. These alkyd resins may have originated from antifouling paint coatings of fishing boats; however, there was no additional evidence to prove this hypothesis. Furthermore, PMMA resins prevailed in the northwestern region of the German Bight with a concentration of 1032 ppt and accounted for 83% total polymer types. Given that the highest amount of PMMA was found within a major shipping lane, these PMMA resins were considered to originate from binders used in antifouling paint on ships [19]. Moreover, some microplastics discovered in European shelf seas in the North Atlantic Ocean were found to be Cu-based antifouling formulations, and other metals such as Pb and Fe therein were also related to antifouling paints from navigating ships [20]. In light of the higher chemical toxicity of paint particles than similarly sized microplastics of metallic additives [16], the investigation of APPs and marine paint particles is urgently needed, and it is the focus of the present study. Given these characteristics, herein, microplastics and paint microparticles including APPs are called microparticles (MPs).

The sea-surface microlayer (S-SML) is the interface between the atmosphere and sublayer water. MPs in the S-SML can be transferred into the upper marine air through bubbles [21] or sink into the deeper water column or into sediments due to biofouling [22]. Meanwhile, due to the water surface tension and the sticky microgel produced by microbial activity [23], MPs are prone to accumulate in the S-SML. However, due to the relatively high requirements in time, cost, and equipment for collecting samples from the S-SML, compared with the collection of water column and sediments, investigations on MPs in the S-SML are few in Japan and worldwide. For all these reasons, this study focused on MPs in the S-SML that may yield extensive and severe MP pollution. It was noted in relevant investigations where manta trawl nets (330 μm mesh) or neuston trawl nets (300 μm mesh) were used for sampling that large-size MPs (>300 μm) were always detected because smaller MPs less than 330 μm could not be captured. For instance, MPs in surface seawater in the South China Sea were found to be around 0.045 ± 0.093 items/m^3^ when a bongo net (333 um mesh) was used; however, this was changed to 2569 ± 1770 items/m^3^ when a pump was used [24]. In the latter case, smaller-size fractions (<0.3 mm) contributed 92% of the total MPs load [24]. Even though tiny MPs (<50 μm) are significantly abundant [25] and pose a higher risk of being incorporated into organisms’ tissues [26] than larger MPs, few studies identify and evaluate them [27]. Thus, these tiny MPs have been ignored in many studies due to high-tech instrument requirements and time-consuming determination [28], which can lead to a serious underestimation of MP loads in the environment and their corresponding risks [29]. As such, MPs of 10–53 μm size were especially collected and analyzed in this study.

Based on the above theories, we herein make the hypothesis that more MPs may accumulate in the S-SML and along navigation routes, and most of these MPs may be related to antifouling and marine paint. For evaluating the MP pollution status of Osaka Bay in Japan, the present study sought to (1) assess the occurrence and distribution of MP loads at different depths of the water column (S-SML and bulk water), (2) estimate the disparity of MP loads among different sampling zones such as navigation routes, the coastal area, and the bay center, and (3) investigate the abundance, spatial distribution, polymer types, size distribution, and sources of these MPs. This study can be of significance in providing data on MPs’ fate and distribution at a regional or global scale, and it can also provide some theoretical guidance for controlling MP pollution.

## 2. Materials and Methods

### 2.1. Sampling Sites

Osaka Bay, located in western Japan, is connected to the enclosed sea area of Harimanada through the Akashi Strait in the northwest. Eastward water transport always prevails in the Seto Inland Sea due to westerly winds, and significant tidal currents with a complex dynamic mechanism are formed in the Akashi Strait, which leads to the contamination of Osaka Bay by the transport of pollutants through this strait [30]. Meanwhile, cyclonic circulation at Osaka Bay both in summer and in winter can also provide impetus to water conveyance from the Kii Strait in the southeast and finally, to the Pacific [31]. Coupled with domestic and industrial activities from international trade ports, large industrial centers, and coastal industrial cities in Hanshin Industrial Zone around Osaka Bay, this situation has led to the formation of terrestrial and marine plastic debris arising from the whole of Western Japan, which is then exported towards the Pacific.

**Figure 1:**
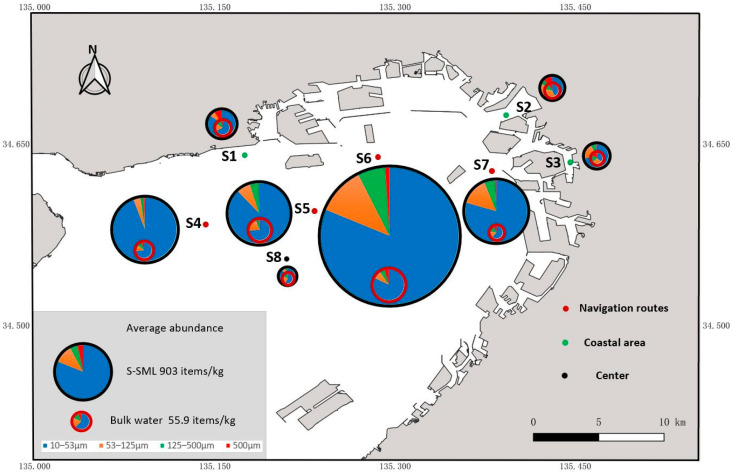
Sampling sites and spatial distribution of average MPs’ abundance.

It should be noted that the water circulation is quite significant in Osaka Bay with a tidal range about 1 m every day; as such, the MPs’ abundance in the estuary is directly related to the tidal fluctuation [32]; for this reason, the estuary was excluded from the sampling sites as much as possible in this research, just apart from site 2 that locate at estuary of Yodogawa. Considering that the purpose of this study was to investigate the discrepancy in MPs’ abundance among different marine zones, in total, the sampling sites included 3 sampling zones (Figure 1 and Table S1); S1, S2, and S3 are located in the coastal area, S4, S5, S6, and S7 are located along shipping navigation routes, and S8 is the center of Osaka Bay. This design allowed for the testing of our hypothesis that MPs’ abundance along the navigation routes is significantly higher than that in the coastal area and the bay center.

### 2.2. Field Surveys

All water samples were collected from September to June from 2021 to 2023 (Tables S1 and S2). In order to more accurately reflect the average MPs’ abundance at each sampling site, sampling was usually repeated 2–4 times. The repeated samplings were conducted on different dates, and it is impossible to ensure that the currents, tides, and meteorological conditions on these different dates were under the same level. In this sense, repeated samplings at the same site can yield results under different sea and meteorological conditions, which can reflect the average MPs’ abundance with the local characteristics of Osaka Bay.

The S-SML was sampled by an electric rotating drum sampler (RDS) with an acrylic cylindrical drum (diameter 0.25 m, width 0.45 m, surface area 0.353 m^2^). A total surface area of 212 m^2^ of seawater was collected at 20 rotation per min for 30 min. Meanwhile, bulk water below 1 m of sea surface was sampled through a pump (SK-62510, Koshin, Kyoto, Japan) as a contrast substrate. Approximately 10 L of seawater samples (S-SML and bulk water) was collected for each site.

Both S-SML and bulk water samples were filtered through a stainless-steel sieve cascade (φ10 cm) (JIS Z 8801, Tokyo Screen, Tokyo, Japan). For the first filtration, the sieve was equipped with a 500 μm mesh-size filter, the subsequent filtration was performed with a 125 μm mesh-size filter, and the final filtration with a 53 μm mesh-size filter; then, the water was returned to a 10 L tank. The retained MPs on these sieves with separate size ranges of >500, 125–500, and 53–125 μm, respectively, were rinsed into bottles with the aid of ultrapure water. As a result of the sieving process, the remaining MPs in the tank water samples were less than 53 μm. Bottles and tanks were sealed and stored on the experimental ship, then carried back to the laboratory.

### 2.3. Laboratory Pretreatment

MPs in bottles (with size ranges of 53–125, 125–500, and >500 μm) were filtered through a PTFE filter (POPMF-1000, 10 μm, Wintec, Kobe, Japan) with a diameter of 47 mm, and MPs in tanks (with size of <53 μm) were filtered through a PTFE filter (POPMF-1000, 10 μm, Wintec, Kobe, Japan) with a diameter of 142 mm. The filters with retained MPs were kept in Petri dishes and dried at 60 °C for 12 h; then, they were soaked in 30% H_2_O_2_ solution for 1 week, to digest organic matter. For removing skins and shells of algae and microbe coverings on samples, MPs with size ranges of 10–53, 53–125, 125–500, and >500 μm on the filters were washed down and filtered through a nylon filter with a mesh size of 10 μm (#500/520, Tantore, Aichi, Japan), 48 μm (No. 305T, AS ONE, Osaka, Japan), 108 μm (No. 150T, AS ONE), or 500 μm (NB-34, AS ONE), respectively. Especially for MPs of size 10–53 μm, this vacuum filtering process lasted for more than 40 min to fully air-dry and fix algae shells on the nylon filter, so that MPs could be completely separated from them. The MPs retained on these nylon filters were rinsed apart from the fettered algae shells and filtered through a PTFE filter (H100A013A, ADVANREC, Osaka, Japan, 1 μm, 13 mmφ). Since MPs with a size of 10–53 μm could clog filter pores when in abundance, they were washed with 100 mL of ultra-pure water and filtered five times with five separate filters, rather than just filtered once. Finally, all filters were dried at 60 °C for 12 h and used for microscopic observations. All filtering processes operated in a vacuum filtration system. MPs were rinsed by ultrapure water, and filters were dried in a dryer (D-450 FA, AS ONE, Osaka, Japan). The relevant process of water sampling and laboratory treatment is shown in Figure S1.

### 2.4. Detection and Identification of MPs

MPs on filters were observed and counted by an infrared microscope (AIM-9000, SHIMAZU, Kyoto, Japan) and their polymer types were analyzed and identified by a Fourier transform infrared spectrometer (FTIR: IRTracer-100, SHIMADZU, Kyoto, Japan). For MPs with a size >500, 125–500, and 53–125 μm, a ¼ square of the filter was chosen, the plasticlike particles retained there were detected, and MPs’ abundances and corresponding polymer types were measured and recorded. For MPs with a size of 10–53 μm, due to their high concentrations and their laborious counting, the particles were counted in three squares of the filter paper only, each of which was randomly assigned from a center, middle, and outer zone of every PTFE filter [33]. One square was 1.6 × 1.8 mm^2^, and three squares were approximately 1/9 of the total filtered area (Figure S2). MPs with a size of 10–53 μm on all five filters were counted repetitively in that way. The polymer type of each MP could be identified by measuring its transmittance and matching its IR spectrum to the one in the FTIR polymer spectrum library with a matching degree higher than 70%. All the polymer types found in this study were acrylic resins, mainly represented by polymethyl methacrylate (PMMA), polypropylene (PP), polyethylene (PE), polyester (PET), polystyrene (PS), nylon (PA), polyvinyl chloride (PVC), and others.

To exclude contamination from environmental MPs such as nylon filters, PS bottles, and PE tanks, ultrapure water was used as the blank water sample to repeat the above processes of treatment and filtration. No MPs were detected in the blank samples; as such, the contamination from the equipment was negligible.

### 2.5. Data Analysis

QGIS 3.38.0 (QGIS.ORG, https://qgis.org/ja/site/, accessed on 3 November 2023) was used for mapping sampling sites in the Osaka Bay shown in Figure 1. The differences in MPs’ abundances in the S-SML and the corresponding bulk water below were assessed through the Mann–Whitney U test. In addition, the differences in MPs’ abundance (containing all polymers or PMMA) in the S-SML or in bulk water among different sampling zones were analyzed by a one-way ANOVA followed by Tamhane’s T2 post hoc test. A simple linear regression was carried out to test the linear relationship between MPs’ abundance and size. A Pearson correlation analysis was conducted to measure the correlation between MPs’ abundance and other factors including polymer types, size ranges of MPs and depth, pH, salinity, temperature, and TOC and DOC of water sampling. MP sources were summarized by the method of principle component analysis (PCA). The correspondence analysis was conducted to reveal the relationship among abundances of MPs with every polymer type, depth, sampling zone, and size range. All the data analyses were performed with SPSS 19.0 (IBM, New York, NY, USA) and Origin 2021 (OriginLab, Northampton, MA, USA). The curves of polymer spectra were visualized by EZ OMNIC. All the figures were drawn in Excel and Origin 2021.

### 2.6. Error and Accuracy

All experimental steps were strictly conducted according to previous relevant standards to ensure a high accuracy of results, but inevitable errors still existed. For site 5, the GPS coordinates were 34°35.639′ N 135°14.259′ E on 15 September 2021 and 34°35.602′ N 135°14.030′ E on 5 September 2022, respectively. The distance between the two GPS coordinates was about 358 m. Because the berths of experimental ship could not be exactly ensured at the same coordinate of latitude and longitude every sampling time, slight differences of actual geographical location for one site at every sampling time are always inevitable. MPs’ abundance at one site among different sampling times may be affected by tidal range and current. In addition, as all MPs on filter could not be counted simultaneously, there must have been some errors in estimating the total MPs’ abundance. Moreover, some MPs that had undergone long-term weathering always showed a low peak of FTIR spectra, which made their matching degree less than 70%. These particles always resulted in a “no-polymers” matching and were excluded from the identification of MPs; this may have led to an underestimation of the real MPs abundance.

## 3. Results and Discussion

### 3.1. Spatial Distribution of MPs

As shown in Figure 1, MPs in the S-SML and bulk water in Osaka Bay exhibited higher concentrations along navigation routes (S4, S5, S6 and S7) than that in the coastal area (S1, S2 and S3) and at the center of the bay (S8). Especially in the S-SML, MPs’ abundance along navigation routes was significantly higher than that in the coastal area (one-way ANOVA Tamhane’s T2 post hoc test, *p* < 0.05) and in the center (one-way ANOVA Tamhane’s T2 post hoc test, *p* < 0.05).

The average abundance of MPs in the coastal area was 402 ± 179 items/kg (ranging from 104 to 575 items/kg) in the S-SML, about 10 times higher than the 43 ± 16.4 items/kg (ranging from 21.8 to 66.6 items/kg) in bulk water. In addition, the average abundance of MPs at S1, S2, and S3 was 457 ± 7.78, 373 ± 243, and 390 ± 255 items/kg in the S-SML and 48.7 ± 4.67, 43.2 ± 16.4, and 38.5 ± 24.5 items/kg in bulk water, respectively. The small difference in MPs’ abundance both in the S-SML and in bulk water between S1, S2, and S3 indicated a similar MP pollution distribution in the coastal area.

The average abundance of MPs along navigation routes was 1360 ± 1050 items/kg in S-SML, about 20 times higher than of the 68.3 ± 49.3 items/kg in bulk water. The maximum abundance among all sampling sites was found at S6, with 2310 ± 1390 items/kg in the S-SML and 108 ± 73.3 items/kg in bulk water. Other sites along navigation routes had similar MP loads. The average abundance of MPs at S4, S5, and S7 was 1070 ± 1120, 1020 ± 662, and 1040 ± 773 items/kg in the S-SML and 56.6 ± 16.2, 71.2 ± 44.8, and 43.9 ± 47.1 items/kg in bulk water, respectively. Most previous studies have shown that MPs’ abundance in the coastal area is always higher than that in water areas far from shore [17,34,35,36]; however, in this study, although navigation routes were far from shore, MPs’ abundance therein was significantly larger than that in the coastal area. We believe that MPs newly generated along navigation routes had replaced the ones lost due to long-distance transportation from nearshore to further sea areas; this caused an MP abundance at navigation routes both in the S-SML and bulk water that was about 2–4 times larger than in the coastal area. This result suggests that the number of marine-based MPs generated during navigation far exceed the number originating from land-based MPs, which is quite different from the widely cited 80% terrestrial-to-20% marine-based MP debris ratio for MPs. Similar results to ours were found in a study in the German Bight [19], which also corroborates that these MPs may result from the generation of particles from antifouling and marine paints during the navigating process.

As shown in Figure 1, the average abundance of MPs at the center of the bay (S8) was 261 ± 240 items/kg in S-SML, about nine times higher than the 33.1 ± 17 items/kg (ranging from 23.4 to 50.9 items/kg) in bulk water. These numbers were the lowest among all sampling sites, and they also reflected the decrease and loss of MPs from nearshore to offshore.

Figure 1 shows the average abundance of MPs among all different sampling times for every sampling site. However, notably, as shown in Figure S3, the fluctuation in MPs’ abundance among different samplings was extraordinarily large in the S-SML both at S4 (ranging from 342 to 1070 items/kg with a CV of 105%) and S8 (ranging from 62.7 items/kg to 528 items/kg with a CV of 92%). This result may be due to the exchange of seawater through the Akashi Strait in the western side of Osaka Bay. Freshwater inflow from all rivers on lands to the Seto Inland Sea has been reported to be approximately 14 km^3^/year (38 × 10^6^ m^3^/day) [37], 91% of which corresponding to seawater flows through the Akashi Strait from Harimanada [38]. As such, original MPs accumulated at S4 and S8 might be diluted by freshwater flows and then transported to the eastern part of Osaka Bay, which led to the relatively lower MPs’ abundances. In total, among all sampling sites, MPs’ abundance in the S-SML always showed larger CV values and presented more intense fluctuation than that in bulk water. At the bay center, where all seawater circulation can flow through, the CV value was 110% in the S-SML and 36% in bulk water, respectively. Thus, we can formulate a hypothesis that in the highly mobile and easily diffused S-SML, MPs’ abundance constantly changes, and a large fluctuation is noted in the process of long-distance transportation and migration.

### 3.2. Size Distribution of MPs

**Figure 2:**
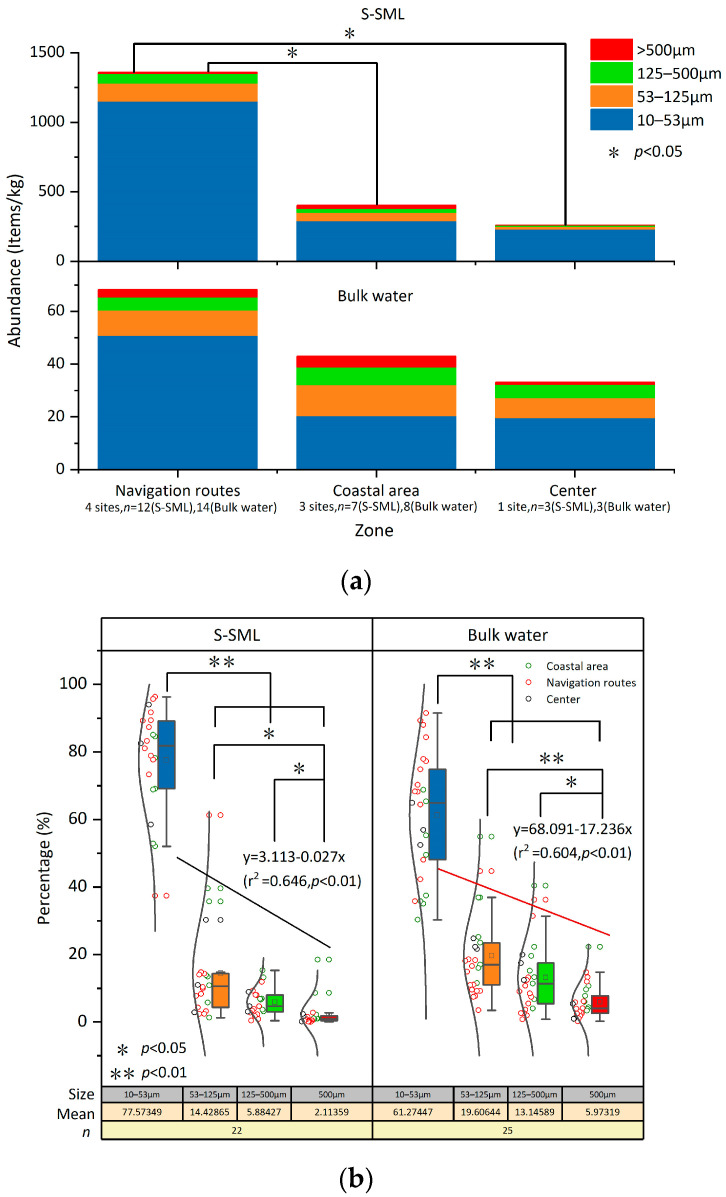
Average abundances of MPs with different size ranges at different sampling zones (**a**) and percentage of MPs with different size ranges at all sampling sites in total (**b**).

MPs were classified in four size categories: 10–53, 53–125, 125–500, and >500 μm, which accounted for 81.2%, 11.1%, 4.45%, and 3.25% in the S-SML, and for 62.2%, 19.8%, 12%, and 6% in bulk water evenly among all sampling sites, respectively. When distinguishing between substrates, as shown in Figure 2, the tiny MPs (10–53 μm) accounted for 72.1%, 84.6%, and 88.5% of all MPs in the coastal area, along the navigation routes, and at the bay center, respectively, in the S-SML. In contrast, the tiny MPs accounted only for 47.4%, 74.4%, and 59.5% for these three zones, respectively, in bulk water. A higher proportion of tiny MPs (10–53 μm) in the S-SML may result from the accelerated fragmentation of larger MPs, because weathering forces including wind, biofouling, and UV irradiation are always stronger and more frequent in the S-SML than in deep water [33]. Thus, larger MPs in the S-SML always undergo a more intensive weathering and break into tiny MPs more rapidly. In addition, MPs less than 100 μm in the water column are easily captured by hydrophobic substances such as grease and microbubbles, which then move upwards [39,40], and this may also lead to an increase in tiny MPs’ abundance in the S-SML. It is noteworthy that these exceptionally well floating tiny MPs (10–53 μm) in the S-SML tend to sink once fouled by algae and invertebrates [2], thus they are transported to the subwater and diffuse the pollution. Hence, further studies should focus on mechanisms of vertical transport of MPs that can be affected by both intrinsic MP buoyancy [41] and hydrodynamic characteristics, including stratification, plume front, and turbulence as well [42]. It should be mentioned that the vertical mixing of plastic particles is probably controlled by wind-induced turbulence, and this leads to a preferential removal of heavier and smaller plastic particles from the sea surface, so traditional surface measurements may have severely underestimated the total plastic content [43]. However, it has been verified that MPs with a size of 10, 100, and 1000 μm maintain their maximum concentration in the top layer of <1 m [44]. Therefore, in this study, MPs in bulk water under 1 m from the sea surface may reflect the relatively real MPs’ abundance. In addition, even if the S-SML is disrupted by wind turbulence, it can still be rapidly reformed without depletion of organic matter within 1 min [23]. Therefore, the influence of turbulence on MPs’ abundance in the S-SML can also be excluded in this study.

As shown in Figure 2b, both in the S-SML and bulk water, as MP sizes increased, their relative abundances decreased (simple linear regression, r^2^ = 0.646, *p* < 0.01 in the S-SML and r^2^ = 0.604, *p* < 0.01 in bulk water, respectively), which was mostly consistent with previous research results [11]. MPs with a size >500 μm were significantly fewer than MPs of other sizes (one-way ANOVA Tamhane’s T2 post hoc test, *p* < 0.05). MPs with a size of 10–53 μm were the most abundant compared to MPs of other size ranges (one-way ANOVA Tamhane’s T2 post hoc test, *p* < 0.01) both in the S-SML and in bulk water. MPs originating from coastal areas always showed high loads in size ranges larger than 53 μm; this may be because these large MPs were newly generated and were not yet weathered and broken into smaller fractions. Among the tiny MPs with a size of 10–53 μm, higher MP loads were mainly along navigation routes rather than in the coastal area and at the bay center. These tiny MPs with larger specific surface areas have increasing sorption capacities, and they can adsorb more pollutants, including persistent organic pollutants (POPs) and endocrine disrupting chemicals, once ingested by organisms, or transported by circulation over long distances. As such, the threats to marine ecosystems of these tiny MPs are multiplied.

### 3.3. Polymer Composition of MPs

Approximately 11 kinds of polymer types of MPs were detected in Osaka Bay, i.e., acrylic resins mainly represented by polymethyl methacrylate (PMMA), polypropylene (PP), polyethylene (PE), polyester (PET), polystyrene (PS), nylon (PA), polyvinyl chloride (PVC), polyurethane (PU), polycarbonate (PC), ethylene-vinyl acetate copolymer (EVA), and epoxy. The corresponding spectra and pictures observed by FTIR are shown in Figures S4 and S5. Since the abundances of PU, PC EVA, and epoxy were relatively small, these four kinds of polymer types were grouped and defined as “others” in this study. Polymethyl methacrylate (PMMA), the most important type of acrylic polymer, is also called acrylic resin [45]. Besides PMMA, other kinds of acrylic polymers were also detected with relatively less abundance through FTIR, and they were also classified as acrylic resin (PMMA).

As shown in Figure S6, PMMA accounted for the highest proportions of MPs in both the S-SML and bulk water, with 95.1% and 45.6%, respectively, followed by PE, PP, PS, PA, PET, others, and PVC, which accounted for 1.22%, 1.13%, 0.851%, 0.632%, 0.591%, 0.372%, and 0.104% in the S-SML, and 16%, 12.8%, 6.99%, 6.56%, 5.88%, 4.27%, and 1.9% in bulk water, respectively. In addition, PMMA accounted for 96.8% in the S-SML and 49.8% in bulk water among all MPs with a size of 10–53 μm.

**Figure 3:**
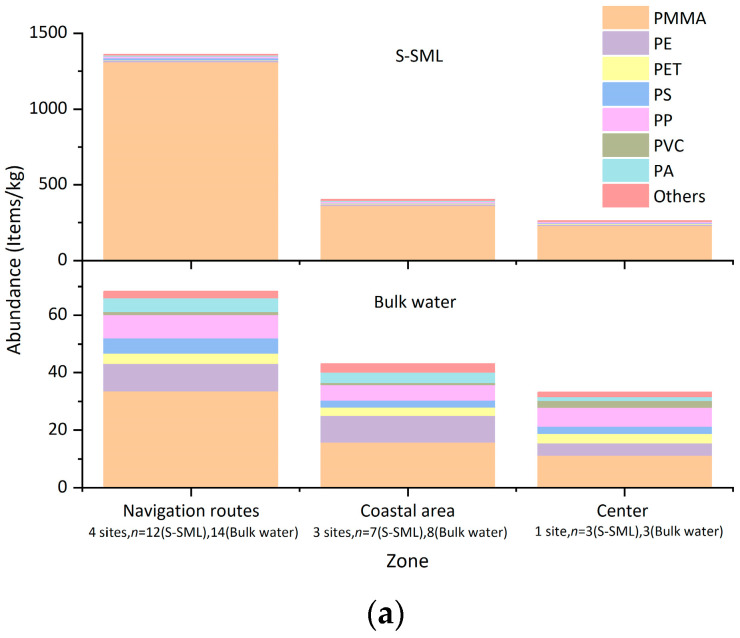

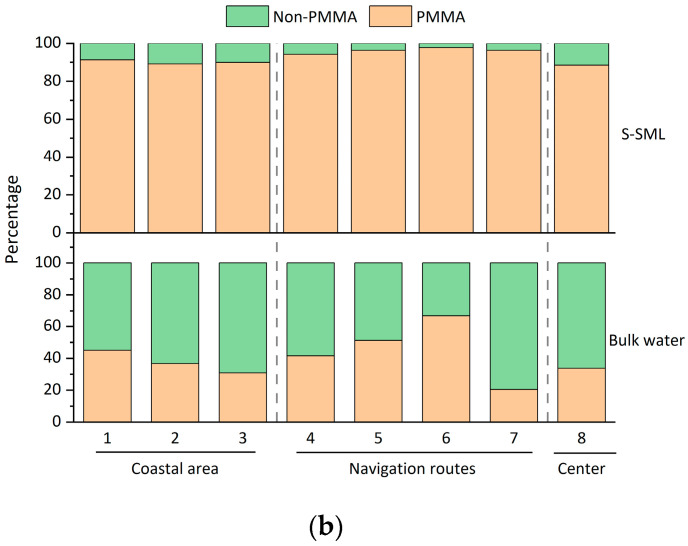
Average abundances of MPs at different sampling zones with different polymer types (**a**) and percentage of MPs with and without PMMA in total (**b**).

As shown in Figure 3, in the S-SML, PMMA dominated all polymer types and accounted for 96.5% (1310 items/kg), 89.9% (362 items/kg), and 88.5% (230 items/kg) along navigation routes, in coastal areas, and at the bay center, respectively. In bulk water, although the percentage of PMMA declined, it was still larger than that of other polymer types and accounted for 49.2% (33.6 items/kg), 36.8% (15.8 items/kg), and 33.7% (11.2 items/kg) at the above sampling zones, respectively. Although the relative density of PMMA is larger than that of the seawater, it still accumulated in the S-SML in large quantities, which can be attributed to the capture capability of the S-SML’s tension.

These abnormally high abundances of PMMA were different from the results in other relevant studies which revealed that PP and PE were dominant polymer types in aquatic environments [46]. According to the conclusion of a similar study at the German Bight, PMMA predominated in local major shipping lanes and navigation routes [19]. This study also suggested the antifouling and marine paints on ship hulls were plausible sources for this pollution.

### 3.4. PMMA from Marine and Antifouling Paints

Acrylic polymers such as PMMA stand out among organic coatings because of their nonwettability, chemical inertness, environmental stability, and photostability [47]. Antifouling paint applied on the surface of the hull bottom can prevent the adhesion of fouling organisms and improve speed and reduce fuel consumption for ships. Highly toxic biocides have been incorporated in binders and then are released into water gradually to accomplish antifouling. The use of acrylic resins as binders in antifouling paints can be traced back to 1955 for insoluble matrix or contact paints and to 1974–1985 for self-polishing paints containing tin (TBT-SPC) [48]. Meanwhile, according to the formulas of antifouling paints in Japan, acrylic resins including PMMA were also used as binders [49]. For insoluble matrix or contact antifouling paints, their binders are insoluble in seawater, and pores can be left empty after biocides diffuse out of paint layers, then seawater can spread through these pores and dissolve biocides in deeper binders [48], which may accelerate the abrasion of the honeycomb structure of residual binders with seawater, finally leading to the exfoliation of these acrylic resin fragments without further hydrolysis in a relatively static water column. In addition, for self-polishing antifouling paints that are mainly composed of (meth)acrylic polymer binders [50], as pigments and biocides dissolve faster than the binder hydrolysis, a leached layer is formed at 10–30 μm behind the binder front [51]. Biocides can then be released through a leached layer continuously accompanied by the binder hydrolysis, through which the self-renewing of antifouling paint surface can be achieved [52]. Although (meth)acrylic binders can be soluble in water by hydrolysis of the silyl ester bond [50], for most self-polishing antifouling paints, their antifouling ability mainly depends on the shear of seawater flow and is therefore poor in static seawater [53]. Hence, once the hydrolysis of acrylic binders is prevented by biofilm-covered surfaces, APPs from shipyards by wind and rain runoff may persist in marine environments with a low water flow. In particular, during the navigating process, as hydrolysis occurs, more hydrophilic ester bonds (-COO-) are exposed on the paint surface, which makes the resin on the paint surface brittle and easy to peel off [54]. The friction and collision between the hull and seawater may exacerbate the exfoliation of these resins. Once detached from the hull bottom, these resin fragments can remain in a relatively static water environment without further hydrolysis for a long time, then break into APPs due to UV weathering and biodegradation. By contrast, ships locating in coastal areas are always in a static water environment, and the antifouling paints cannot be hydrolyzed due to the shear of seawater flow; thus, the resins on the surface of paints may not delaminate from the hull bottom. This leads to a lower APPs’ abundance in the coastal area compared to navigation routes. Actually, APPs detected in the coastal area mainly come from ship building and boat cleaning at shipyards.

Besides antifouling paints, PMMA is also widely used in marine paints that are applied on the outboard and superstructure of hulls to maintain beauty and gloss. It is noteworthy that acrylic paints are prone to dehiscing and cracking at low temperatures [55], and it has been verified that some acrylic paints become brittle at 5 °C with 50% relative humidity (RH) and even at 13 °C with a lower RH [56]. Therefore, these PMMA marine paints on upper hulls are also prone to becoming brittle and then falling off from the ship structure when navigating, especially in winter, which can result in abundant white PMMA fragments and fiber residues (shown in Figure S4) in the S-SML.

Although MPs in this study were mainly in the shape of fragments, large quantities of PMMA fibers were found in the S-SML at S2, S3, S6, and S7, and their abundances were far larger than that of PMMA fragments. Significantly, most of these PMMA fibers were in the size range of 53–125 μm; therefore, they may have originated from UV weathering and wave fragmentation of MPs with a size of 125–500 μm and >500 μm. If not quickly exchanged with open ocean waters, these PMMA fibers might continuously be broken down and become smaller and more abundant, especially in the local semienclosed waters at S3 and S7. Thus, PMMA fibers may be deemed to be a special shape of disintegrating vectors through which PMMA fragments decompose from larger sizes to smaller sizes.

**Figure 4:**
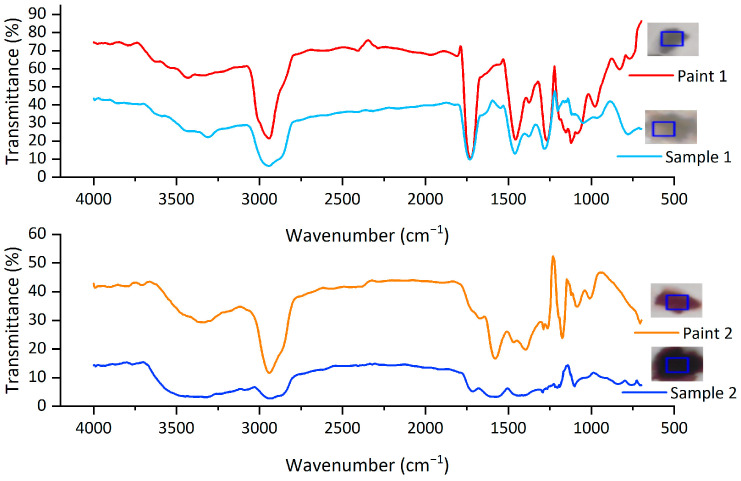
FTIR spectra of polymer types from MPs in Osaka Bay and selected marine and antifouling paints.

To further verify whether PMMA and other acrylic polymers in this study derive from antifouling and marine paints, two kinds of paints (as shown in Table S3) with acrylic polymers as binders were chosen to match with MP samples found in Osaka Bay. Paint 1 was used for the outboard and superstructure of hulls to maintain beauty and gloss. As shown in Figure 4, the FTIR spectra of sample 1, which belonged to a PMMA fragment in the S-SML, matched with that of paint 1. For sample 1, a peak at 2942 cm^−1^ showed the stretching vibrations of C-H of methylene, a peak at 1731 cm^−1^ attributed to the bending of saturated C=O-, and the asymmetric stretching vibration of ether C-O-C showed between 1300 and 1000 cm^−1^ and had a peak at 1281 cm^−1^, according to which the polymer type of sample 1 was identified as PMMA. As the FTIR spectra of sample 1 were clearly presented in all PMMA samples and consistent with that of paint 1, these PMMA samples were assumed to originate from marine paints on the upper structures of ships.

Meanwhile, since antifouling paint is one potential source of APPs, the match between MP sample and selected paint 2, one kind of antifouling paint, was conducted to verify this hypothesis. As shown in Figure 4, sample 2, which was one MP fragment in the S-SML, was determined as an acrylic polymer but not as PMMA, and its FTIR spectra showed a significant peak at 2939 cm^−1^ as C-H stretching vibrations, 1714 cm^−1^ as C=O stretching vibrations and 1100, 1204, and 1293 cm^−1^ as C-O-C stretching vibrations, respectively. The FTIR spectra of both sample 2 and paint 2 were consistent, thus pervasive acrylic MPs represented by sample 2 may derive from antifouling paints. Most notably, the main polymer component in acrylic paints is not just confined to PMMA monomer; other acrylic derivative polymers containing acrylate and methacrylate are also widely used in acrylic paints as binders. The spectra peaks of two MP samples in the S-SML were both smaller than those of nonexposed paint samples, which can reflect a higher weathering degree of MP samples that had been floating in seawater for months and years. Therefore, this study is the first to provide empirical evidence of the link between acrylic resins and marine and antifouling paints.

To exclude the possibility that abundant PMMA was generated from the experimental ship, antifouling paints were obtained from the bottom hull of the experimental ship and analyzed by FTIR with a spectral result of polyamide 6:poly(ethylene-co-propylene) 4:1, i.e., a blend of PA 6 and PP-PE with a ratio of 4:1, as shown in Figure S7. Approximate transmittance values at peaks of spectral reflected a similar weathering degree for both MP samples in bulk water and antifouling paints on the experimental ship. As such, some MPs of the polymer type PA 6:PE-PP 4:1 with small abundances could only come from antifouling paint on the experimental ship. Given that PA has been used in the manufacturing of antifouling paint to modify surfaces for reducing bacterial adhesion [57], different from traditional PA fibers from the laundry of textiles and clothes, PA fragments found in this study may also come from marine coatings.

### 3.5. Difference between PMMA and No-PMMA MPs

**Figure 5:**
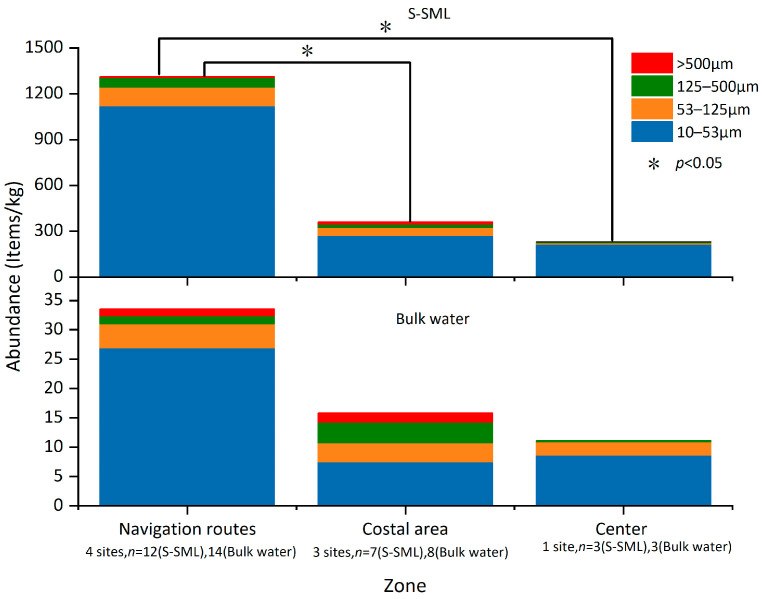
Average abundances of PMMA MPs with different size ranges at different sampling zones.

As shown in Figure 5 in S-SML, the average abundance of PMMA was 1310 items/kg along navigation routes, 362 items/kg in the coastal area, and 230 items/kg at the bay center, respectively, while 33.6 items/kg, 15.8 items/kg, and 11.2 items/kg were found in corresponding zones, respectively, in bulk water. PMMA MPs with a size of 10–53 mm contributed the most in both S-SML (85.4% along navigation routes, 75% in the coastal area, and 93% at the bay center) and in bulk water (80.1% along the navigation routes, 47.2% in the coastal area, and 77.3% at the bay center). Especially in the S-SML, the abundance of PMMA MPs with a size of 10–53 μm along the navigation routes was significantly higher than that in the coastal area (one-way ANOVA Tamhane’s T2 post-hoc test, *p* < 0.05) and at the bay center (one-way ANOVA Tamhane’s T2 post hoc test, *p* < 0.05). Also, the PMMA abundances of all size ranges were higher along the navigation routes than in the coastal area (one-way ANOVA Tamhane’s T2 post hoc test, *p* < 0.05) and at the bay center (one-way ANOVA Tamhane’s T2 post hoc test, *p* < 0.05). However, no significant differences in PMMA abundance were found in bulk water for all sampling zones. The abnormally high PMMA abundance along the navigation routes clearly shows that shipping activities brought about massive acrylic marine and antifouling paint particles and produced large amounts of PMMA MPs with a size of 10–53 μm in the S-SML that may persist along the shipping lanes for months and years.

**Figure 6:**
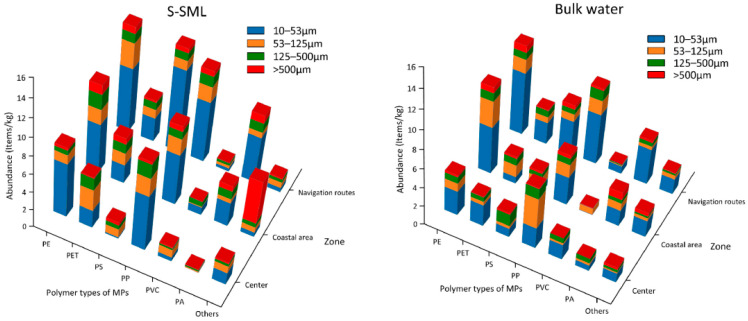
Average abundances of non-PMMA MPs with different size ranges in different sampling zones.

Although the PMMA abundance in the S-SML was significantly larger than that in bulk water, as shown in Figure 6, MPs that did not belong in the PMMA category (non-PMMA) exhibited a similar abundance level of less than 16 items/kg in both S-SML and bulk water. It could also be verified by the correlation analysis in Figure S8, that PMMA abundance was negatively correlated with water depth, while this was not the case for other polymers. As shown in Figure 5, PMMA abundance was the determining factor for the significant differences between S-SML and bulk water for the total MPs’ abundance. It was also a significant factor for the obvious differences in MPs’ abundances between the navigation routes, the coastal area, and the bay center. Compared with PMMA, non-PMMA polymers slightly contributed to all polymer types’ loads both in S-SML and in bulk water. To some extent, the PMMA abundance can even reflect the whole MP loads in the S-SML. As shown in Figure S9, the result of the correspondence analysis showed that PMMA MPs with a size of 10–53 μm along the navigation routes in the S-SML dominated the whole MP loads in Osaka Bay.

Among non-PMMA polymers, PP and PE presented higher abundances than other polymers both in S-SML and in bulk water. In addition, PET, PS, and PA were also more abundant than PVC in general. As shown in Figure S8, for PMMA, PE, PET, PP, PS, and PVC, their abundances increased as their size became smaller.

### 3.6. Sources of Polymer Types of MPs

**Figure 7:**
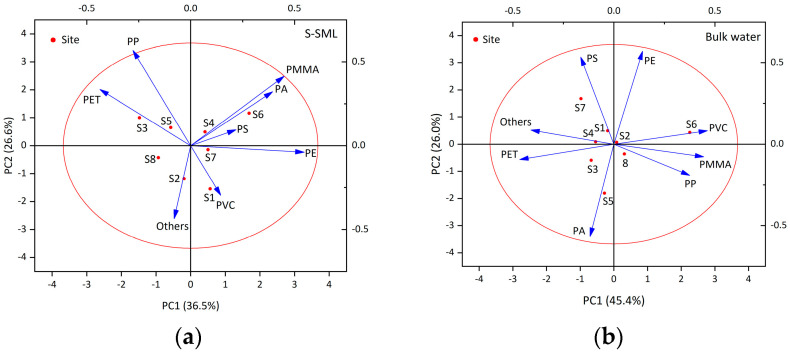
Principal component analysis (PCA) of MPs of all polymer types in S-SML (**a**) and bulk water (**b**).

For assessing the contribution of polymer types of MPs on polymer abundances and on sampling sites, a principal component analysis (PCA) was performed, and the loadings are shown in Figure 7a,b for the S-SML and bulk water, respectively. For the S-SML, two principal components explained 63.1% of the variance in the data, where PC1 contributed 36.5% of the total variance and with positive loadings of PMMA, PA, and PE. PC2 contributed 26.6% of the total variance, with positive loadings of PMMA, PP, and PET. For bulk water, two principal components explained 71.4% of the variance in the data, where PC1 contributed 45.4% of the total variance and with positive loadings of PMMA, PP, and PVC. PC2 contributed 26% of the total variance, with positive loadings of PE and PS.

According to relevant previous research, sources of all polymer types were categorized as shown in Figure S10 [58,59,60,61,62,63]. Except from marine and antifouling paints, PMMA can also originate from terrestrial paints such as those used in building surfaces, road markings, and so on. In addition, PMMA is also the main component of plexiglass, which is widely used in windows, lamps, and dashboards for everyday use, as well as in the manufacturing of airplanes, vehicles, and instruments. Notably, the United States and Japan have made mandatory provisions in their laws that primary and secondary schools and kindergarten buildings must use plexiglass. Furthermore, PMMA microbeads that are used in cosmetics can enter sewage treatment systems directly and can then be released into aquatic environments as primary microplastics. The occurrence of PP and PE is consistent with their huge global production and wide use in modern life, such as plastic bottles, bags, food packing films, fishing gear, caps, and containers due to their low cost and good performance.

In the S-SML, the strong positive loadings of PA, PE and PMMA on PC 1 mainly reflected the emissions of marine and antifouling paints from shipping activities. Site 6 was placed in the direction of the PA loading since it had the highest PA average abundance (14.5 items/kg). In addition, site 6 and site 4—located along navigation routes—were placed in the direction of the PMMA loading because they exhibited the highest PMMA average abundance of 2250 items/kg and 1000 items/kg, respectively, among all sampling sites. Sites 4, 6, and 7 belonging to the navigation routes were aligned on PC1, showing that navigation route sites always generated more paint particles. PC2 was characterized by strong positive loadings of PMMA, PP and PET and showed a linkage with discarded fishing gears generated from fishing activities (fishing nets, ropes, and lines, etc.) according to Figure S10. Site 3 and site 5 were aligned on the direction of PET loading because they exhibited the highest average PET abundance of 9.18 items/kg and 5.64 items/kg. The strong negative loadings of PVC and “others” on PC2 may be due to their relatively low abundance, although PVC had the highest average abundance (3.87 items/kg) at site 1 and “others” had the highest average abundance (11.3 items/kg) at site 2; both site 1 and site 2 are located in coastal areas and they had little relationship with fishing activities. To sum up, the PCA in the S-SML seemed to reveal single marine-based MP pollution sources due to marine activities including shipping and fishing activities.

In bulk water, PC1 showed the positive loadings of PMMA, PP, and PVC indicating diverse MP pollution sources, which may come from domestic consumption such as plastic packages and containers, building, and decorative materials such as PVC pipes and other hard plastics. Site 6 on the direction of PVC loading exhibited the highest PVC average abundance of 2.99 items/kg among all sampling sites. PE and PS’s positive loadings in PC2 indicated the source of raw materials used in mechanical and electrical manufacturing. PA and PET showed strong negative loadings on PC1 and PC2 because these are fibrous MPs generated from clothes, curtains, and carpets during laundry, which had little relationship with package and building decoration. Site 5 on the direction of PA loading had the highest PA average abundance of 13.1 items/kg among all sampling sites. In total, PCA in bulk water may reflect a complex land-based MP pollution source from daily production and life.

### 3.7. Summary of Abundances of MPs in Osaka Bay

MPs were widely distributed in both S-SML and bulk water in Osaka Bay. In the S-SML, the average abundance of MPs ranged from 261 ± 240 items/kg to 2310 ± 1390 items/kg, with an average abundance of 903 ± 921 items/kg. In bulk water, the average abundance of MPs ranged from 33.1 ± 17 items/kg to 108 ± 77.3 items/kg, with an average abundance of 55.9 ± 40.4 items/kg. MPs’ abundance in the S-SML was about 15 times larger than that in bulk water. These are the first abundance-related data available for Osaka Bay.

As shown in Table 1, if 0.45 μm was taken as the minimum size standard, MPs’ abundance in the S-SML in Osaka Bay was much higher than that in Geoje Island [11] and Jinhae Bay in Korea [64] and in Southampton in the UK [65], and MPs’ abundance in bulk water in Osaka Bay was much higher than that in Geojie Island in Korea [11] and Hudson River in US [66]. If 44 μm was taken as the minimum size standard, MPs’ abundance in the S-SML in Osaka Bay was higher than that in Winyah Bay and Charleston Harbor [67] in the US and was similar to that on the Incheon/Kyeonggi Coast in Korea [33], and MPs’ abundance in bulk water was higher than that on the Incheon/Kyeonggi Coast in Korea [33], in the South China Sea [24], in the Terengganu estuary in Malaysia [68], and on the Shore of Rayong in Thailand [69]. Obviously, the abundance of MPs both in the S-SML and bulk water in Osaka Bay was relatively high in relation to worldwide values.

## 4. Conclusions

Our research highlighted the occurrence and distribution of MP pollution in the S-SML in Osaka Bay for the first time. Abundant MPs belonging in the PMMA category were detected both in the S-SML and in bulk water. MPs’ abundances in Osaka Bay were relatively higher than those in the marine areas of other countries. MPs’ concentrations were higher along navigation routes than in the coastal area and at the bay center, wherein MPs with a size of 10–53 μm were the most abundant category among all size ranges. An FTIR spectra analysis verified an abnormally high abundance of MPs, especially PMMA MPs, along navigation routes, suggesting a pollution source from shipping activities. Although MPs’ abundance in the S-SML was significantly larger than that in bulk water, an abundance of MPs with other polymers besides PMMA was similar between the S-SML and bulk water. As such, PMMA was responsible for the large differences in MPs’ abundance among navigation routes, coastal waters, and the bay center. It was also responsible for the significant differences in MPs’ concentration between the S-SML and bulk water.

## Figures and Tables

**Table 1:** Abundances of MPs in surface water in the literature worldwide.

S-SML				Bulk Water			
Size	Country	Site	MPs’ Abundance (Items/L)	Size	Country	Site	MPs’ Abundance (Items/L)
>10 μm	Japan	Osaka Bay (This study)	903	>10 μm	Japan	Osaka Bay (this study)	55.9
>0.75 μm	Korea	Geoje Island ([11])	211	>0.75 μm	Korea	Geoje Island ([11])	0.946
>0.75 μm	Korea	Jinhae Bay ([64])	182	>0.45 μm	US	Hudson River ([66])	0.980
>0.45 μm	UK	Southampton ([65])	75.4	>50 μm	Malaysia	Terengganu estuary ([68])	0.546
>53 μm	Japan	Osaka Bay (This study)	170	>53 μm	Japan	Osaka Bay (this study)	21.1
>50 μm	Korea	Incheon/ Kyeonggi Coastal ([33])	153	>50 μm	Korea	Incheon/ Kyeonggi Coastal ([33])	1.60
>63 μm	US	Winyah Bay ([67])	30.8	>44 μm	China	South China Sea ([24])	2.57
>63 μm	US	Charleston Harbor ([67])	6.60	>75 μm	Thailand	Shore of Rayong ([69])	1.78

## Data Availability

Additional information is available upon request.

## Supplementary Materials

The following supporting information can be downloaded at: https://www.mdpi.com/article/10.3390/jox13040044/s1, Table S1: Sampling date, sites, and water temperature; Table S2: Parameters of water samplings; Table S3: Parameter of two kinds of selected marine paint and antifouling paints; Figure S1: Relevant filtering and dealing process of laboratory treatment; Figure S2: Observing method of MPs on filters; Figure S3: Average abundance of MPs at different sampling sites; Figure S4: FTIR spectra of polymer types of MPs; Figure S5: Pictures of polymer types of MPs; Figure S6: Average percentage of every polymer type of MPs both in S-SML and bulk water; Figure S7: Spectra of APPs on experimental ship and MPs in bulk water; Figure S8: Correlation analysis between polymer abundances and seawater and MPs’ size factors; Figure S9: Correspondence analysis among depth (S-SML and bulk water), abundance of MPs of all kinds of polymers, sizes, and sampling zones; Figure S10: Source of MPs of all polymer types.
