# Supplementary material for: Anthropogenic Microparticles in Sea-Surface Microlayer in Osaka Bay, Japan
Source: J Xenobiot. 2023 Nov 7;13(4):685–703. doi: 10.3390/jox13040044 (PMC10660477; doi:10.3390/jox13040044)
Supplement: Supplementary file 1 [file jox-13-00044-s001.zip › jox-2650575-supplementary.pdf]

Supporting Information

Authors: Mi Zhou, Hirofumi Yanai, Chee Kong Yap, Christina Emmanouil and Hideo Okamura

Manuscript title: Anthropogenic Microparticles in Sea-Surface Microlayer in Osaka Bay, Japan

Table S1 Sampling date, sites and water temperature

Date	Sampling site	Latitude	Longitude	Weather	S-SML water temperature (°C)	Bulk water temperature (°C)
15/9/2021	3	34°38.085'N	135°26.748'E	Sunny	26.6 (10 cm depth)	26.4
	7	34°37.662'N	135°22.834'E	Cloudy	26 (10 cm depth)	26.0
15/11/2021	5	34°35.639'N	135°14.259'E	Cloudy	28.4	28.1
	8	34°33.308'N	135°12.590'E	Sunny	19.3	20.1
	4	34°35.014'N	135°08.558'E	Sunny	19.2	20.3
	1	34°38.310'N	135°10.361'E	Sunny	18.4	18.2
6/12/2021	5	34°35.669'N	135°13.980'E	Cloudy	15.3	15.4
	6	34°38.355'N	135°17.157'E	Cloudy	16.8	16.6
	2	34°40.430'N	135°23.542'E	Sunny	16.9	16.4
11/1/2022	8	34°33.322'N	135°12.575'E	Rainy	10.3	16.0
5/9/2022	5	34°35.602'N	135°14.030'E	Sunny	34.0	30.0
	7	34°37.558'N	135°22.965'E	Sunny	32.5	30.5
	3	34°37.987'N	135°26.942'E	Rainy	31.0	30.2
5/10/2022	5	34°35.553'N	135°13.926'E	Cloudy	23.7	24.6
	6	34°39.121'N	135°16.477'E	Cloudy	24.0	24.8
	2	34°40.298'N	135°25.529'E	Cloudy	24.1	24.5
24/10/2022	8	34°33.848'N	135°12.253'E	Sunny	24.2	22.6
	4	34°34.962'N	135°08.546'E	Sunny	23.7	23.3
	1	34°38.449'N	135°10.496'E	Sunny	22.7	22.7
7/11/2022	7	34°37.472'N	135°22.878'E	Sunny	20.6	20.2
	3	34°38.012'N	135°26.867'E	Sunny	21.6	20.9
	2	34°40.266'N	135°23.535'E	Sunny	22.3	21.2
1/6/2023	4	34°34.962'N	135°08.558'E	Sunny	22.9	21.3
	6	34°38.355'N	135°16.477'E	Cloudy	22.4	21
	7	34°37.662'N	135°22.965'E	Cloudy	21.7	22.7

Table S2 Parameters of water samplings

Date	Sampling site	S-SML volume (kg)	S-SML sampling time (min)	S-SML depth (μm)	Bulk water volume (kg)	S-SML pH	Bulk water pH	S-SML EC (S/m)	Bulk water EC (S/m)	S-SML TOC (ppm)	S-SML DOC (ppm)	Bulk water TOC (ppm)	Bulk water DOC (ppm)
15/9/2021	3	9.5	-	10 (cm)	11.3	7.78	7.81	2.68	3.58	2.84	2.44	2.76	2.30
	7	11.5	-	10 (cm)	10.1	7.95	8.22	3.52	4.43	2.37	2.24	2.32	1.92
15/11/2021	5	10.0	30	47	11.9	8.51	8.43	4.09	4.28	2.90	2.67	2.09	2.08
	8	11.5	30	54	10.7	8.32	8.56	4.89	4.88	3.92	2.81	3.72	2.49
	4	11.8	23	72	11.5	8.11	8.08	4.84	4.85	3.93	1.78	4.06	3.82
	1	11.6	23	71	11.2	7.07	7.98	4.53	4.58	1.96	1.12	3.61	3.51

Date	Sampling site	S-SML volume (kg)	S-SML sampling time (min)	S-SML depth (μm)	Bulk water volume (kg)	S-SML pH	Bulk water pH	S-SML EC (S/m)	Bulk water EC (S/m)	S-SML TOC (ppm)	S-SML DOC (ppm)	Bulk water TOC (ppm)	Bulk water DOC (ppm)
	5	9.7	30	45	11.9	8.50	8.71	3.70	3.77	46.16	29.73	32.34	22.21
6/12/2021	6	9.9	30	46	12.3	8.16	8.11	3.71	3.63	29.19	23.12	40.82	24.55
	2	10.8	23	66	11.8	7.80	7.85	3.62	3.67	21.31	14.14	47.38	30.02
11/1/2022	8	10.8	40	38	12.2	8.60	8.65	3.35	3.77	15.47	13.82	10.80	9.05
	5	9.0	30	42	9	8.99	9.24	5.01	4.90	33.93	11.91	27.89	2.50
5/9/2022	7	9.3	23	56	9.1	9.37	9.09	3.45	4.53	24.21	5.26	14.49	2.74
	3	10.5	20	74	9.8	9.45	9.06	2.87	3.37	20.71	20.47	8.98	7.45
	5	10.3	22	65	12.2	8.39	8.39	4.90	4.87	0.94	0.25	0.53	0.07
5/10/2022	6	10.6	21	71	12.5	8.48	8.39	4.70	4.79	0.96	0.46	0.63	0.13
	2	11.3	22	72	11	8.80	8.31	3.25	4.45	1.86	1.80	1.06	0.44
	8	10.5	20	74	11.8	8.35	8.34	4.90	4.86	0.67	0.53	0.95	0.56
24/10/2022	4	11.0	18	86	11.3	8.41	8.43	4.92	4.90	0.33	0.14	0.29	0.23
	1	9.8	17	81	10.1	8.63	8.53	4.82	4.79	1.01	0.42	0.30	0.25
	7	11.5	18	90	12.1	8.53	8.56	4.53	4.39	1.05	0.73	0.63	0.36
7/11/2022	3	10.2	18	79	11.6	8.47	8.52	3.29	3.71	1.08	0.81	1.02	0.81
	2	11.3	18	88	11.3	8.22	8.23	4.08	4.51	2.58	1.41	1.47	1.40
	4	11.5	26	87	11.1	8.89	8.87	4.56	2.3	20	1.36	19.5	0.96
1/6/2023	6	11.5	19	78	11.6	9.17	9.24	2.92	4	19.4	1.68	19.7	1.09
	7	11.15	18	86	11.85	9.1	8.81	3.5	4.03	21.7	1.4	2.27	1.28

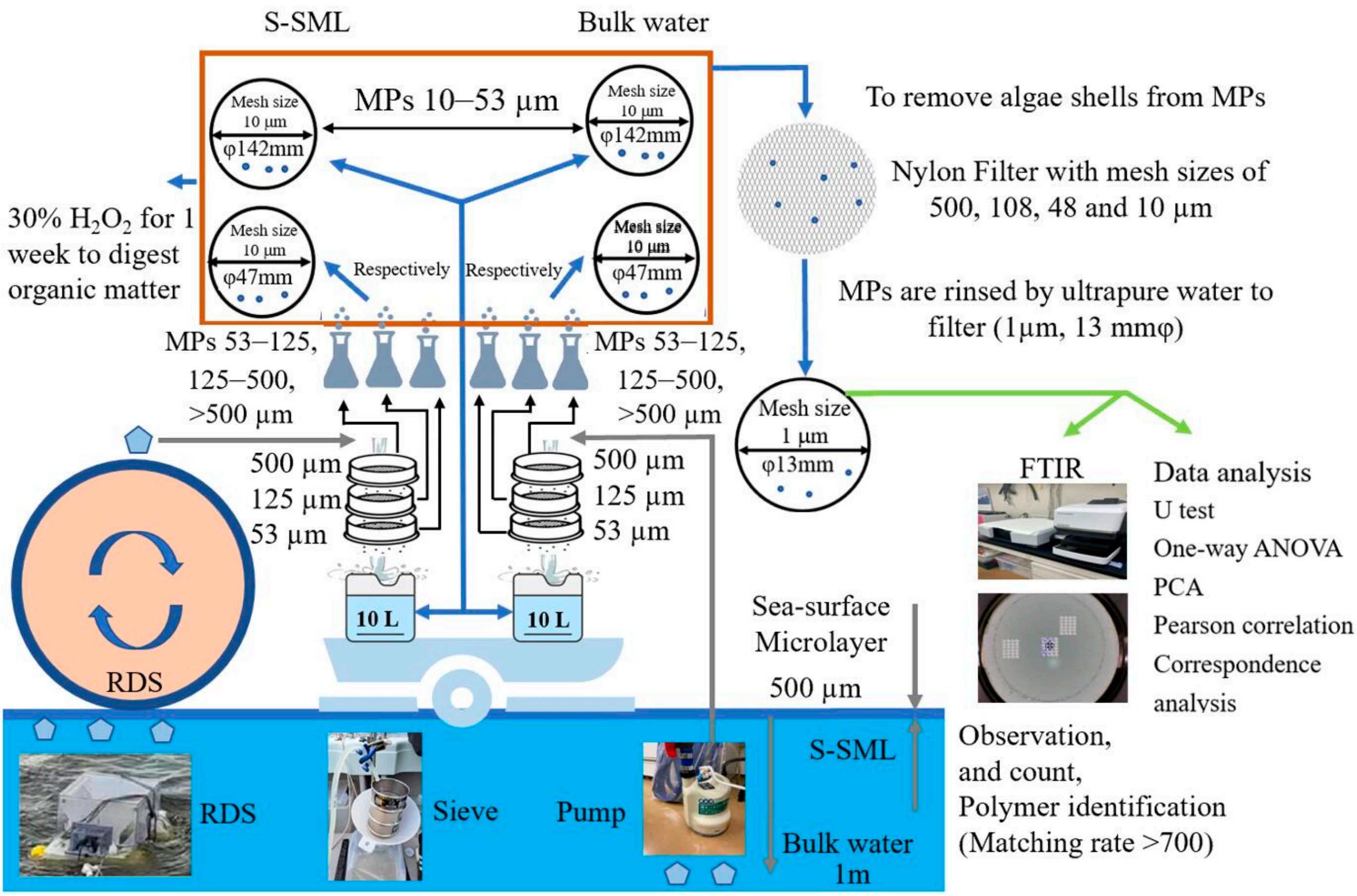

Figure S1 Relevant filtering and dealing process of laboratory treatment

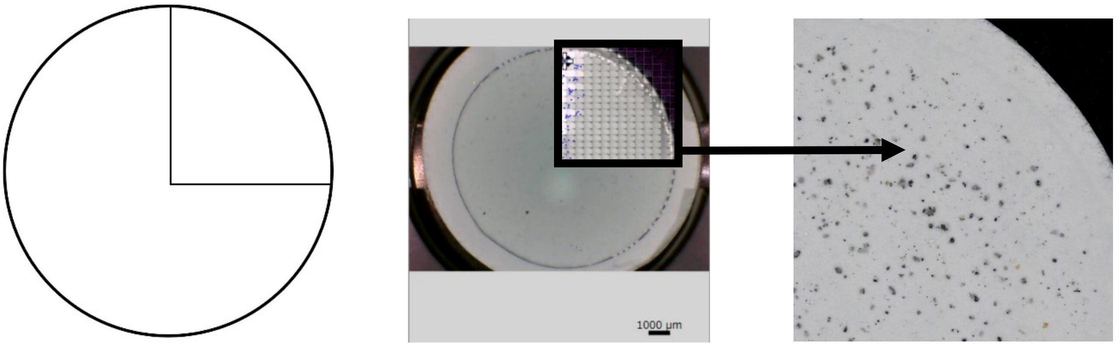

(a)

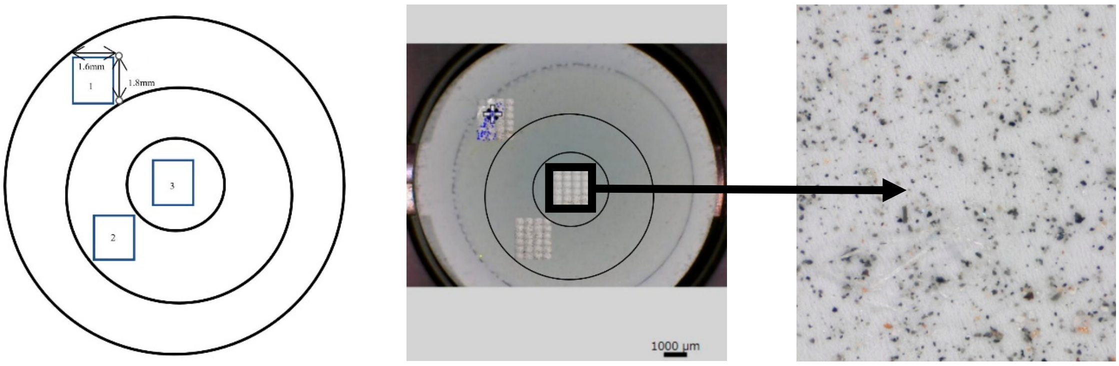

(b)

Figure S2 Observing method of MPs on filters (a) MPs of >500, 125–500 and 53–125 μm (b) MPs of 10–53 μm

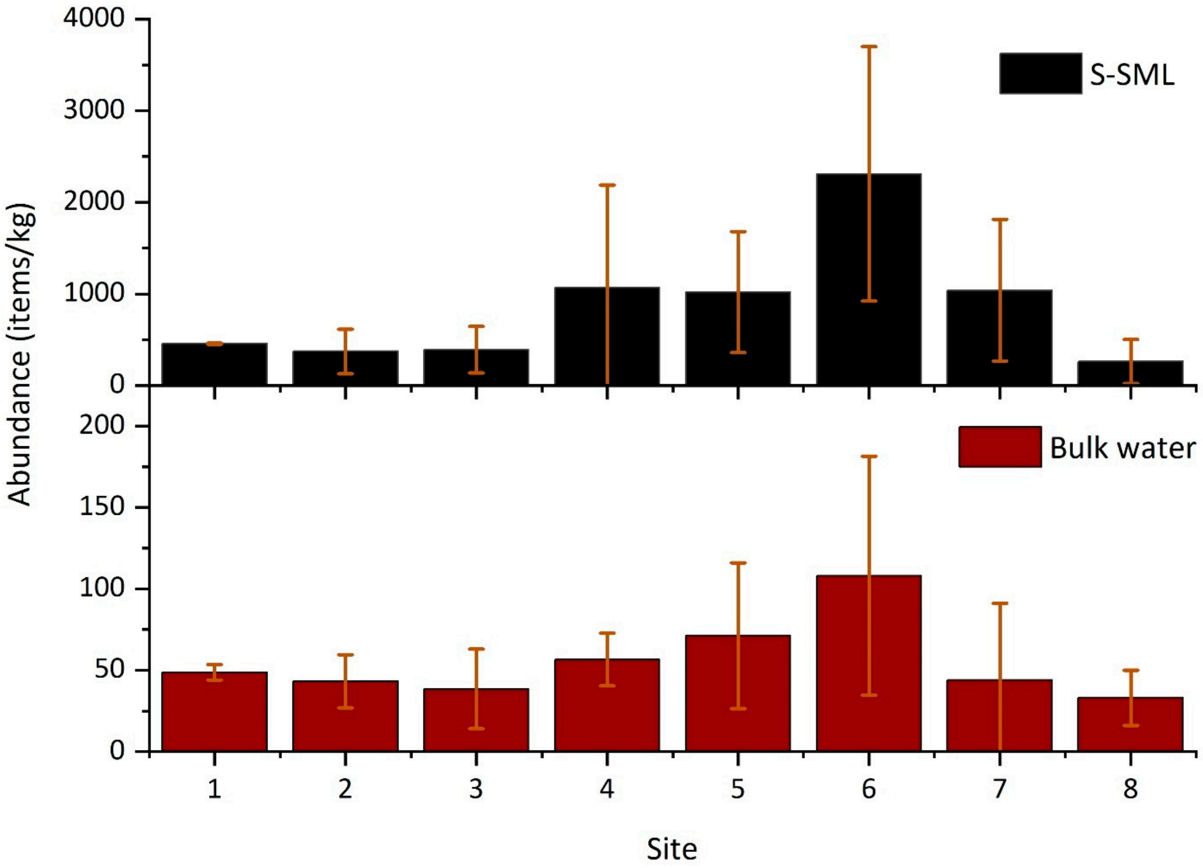

Figure S3 Average abundance of MPs at different sampling sites

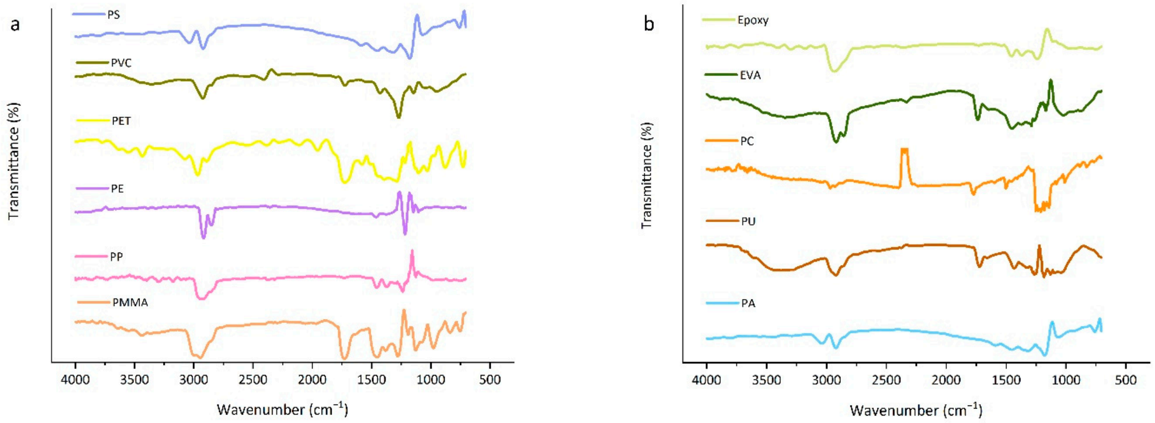

Figure S4 FTIR spectra of polymer types of MPs

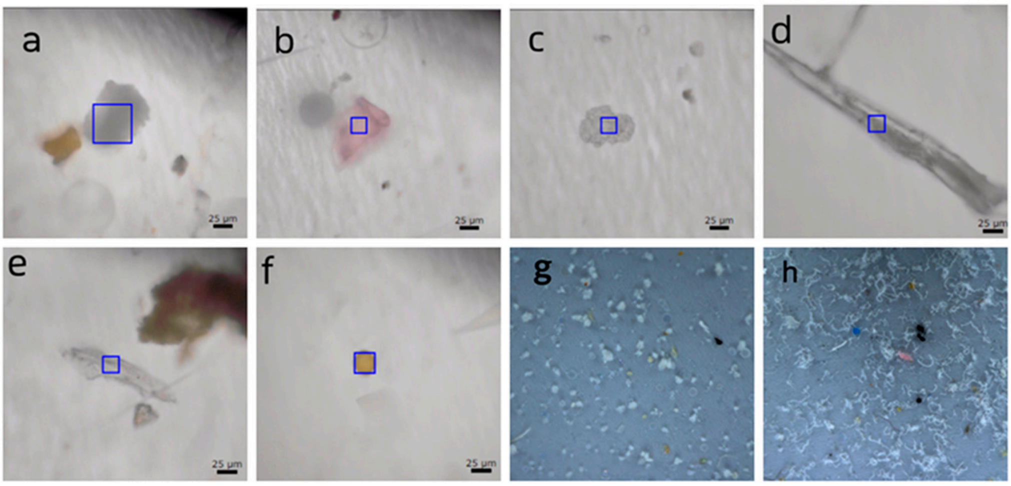

Figure S5 Pictures of polymer types of MPs (a) PMMA (b) PP (c) PE (d) PET (e) PS (f) PA (g) PMMA fragment (h) PMMA fiber

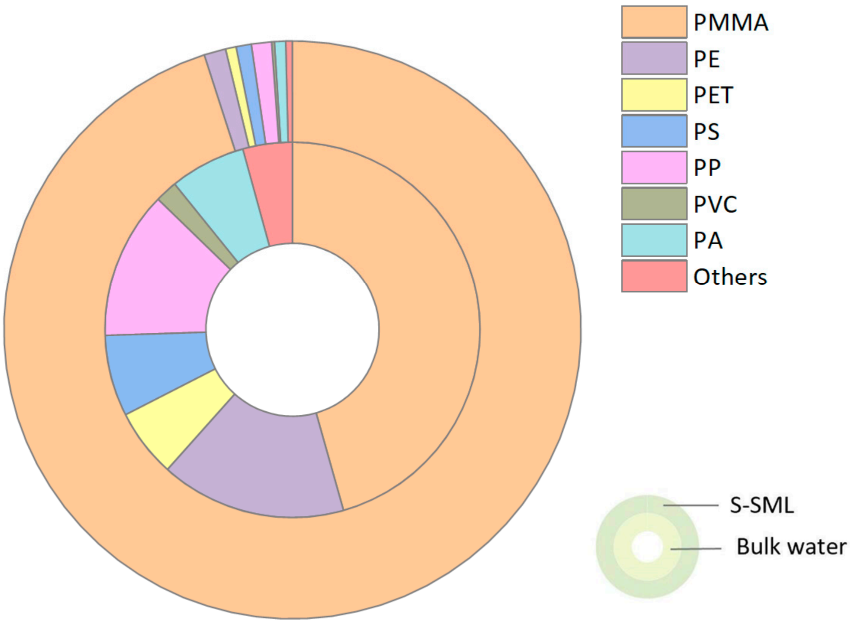

Figure S6 Average percentage of every polymer type of MPs both in S-SML and bulk water

Table S3 Parameter of two kinds of selected marine paint and antifouling paints

No.	Paint name	Manufacture	Polymer Type	Metal
Paint 1	Sea Blue Acepaint for outer side and superstructures	Dainippon Paint Co., Ltd	PMMA	Ti
Paint 2	CCP Cleaning	Kanae paint Co., Ltd.	Acrylic polymer	Cu Zn

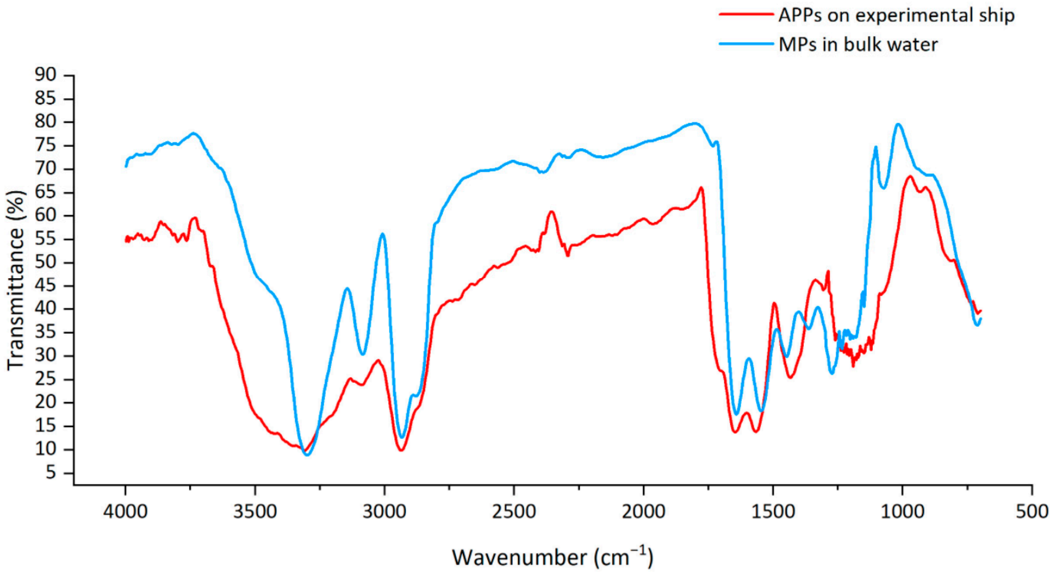

Figure S7 Spectra of APPs on experimental ship and MPs in bulk water

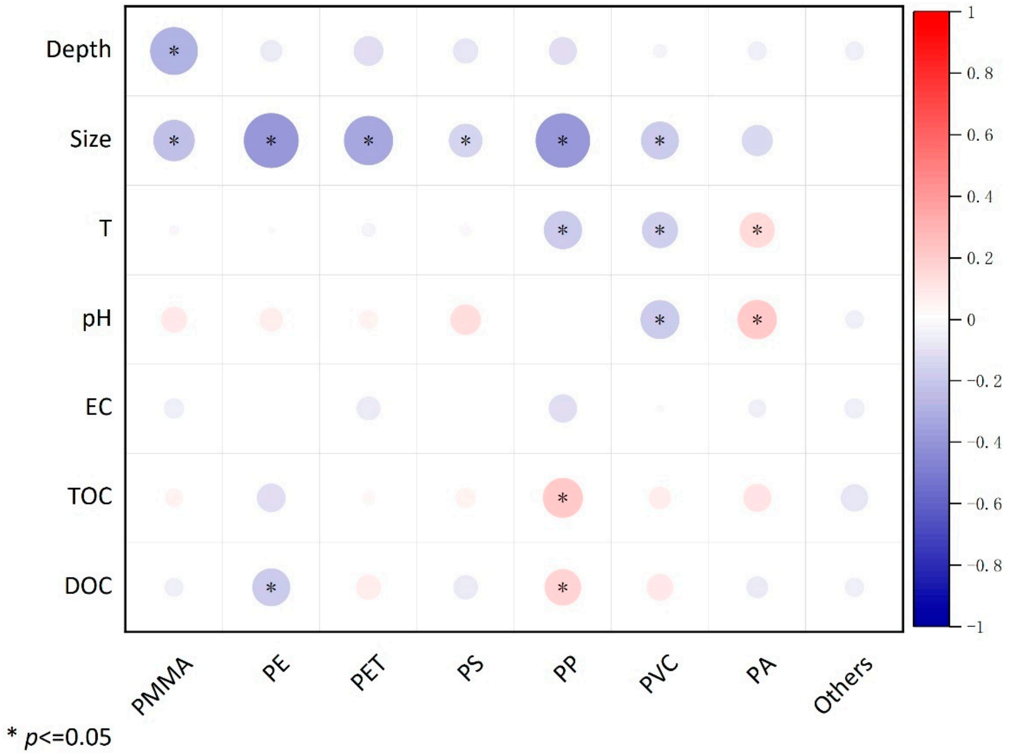

Figure S8 Correlation analysis between polymer abundances and seawater and MPs size factors

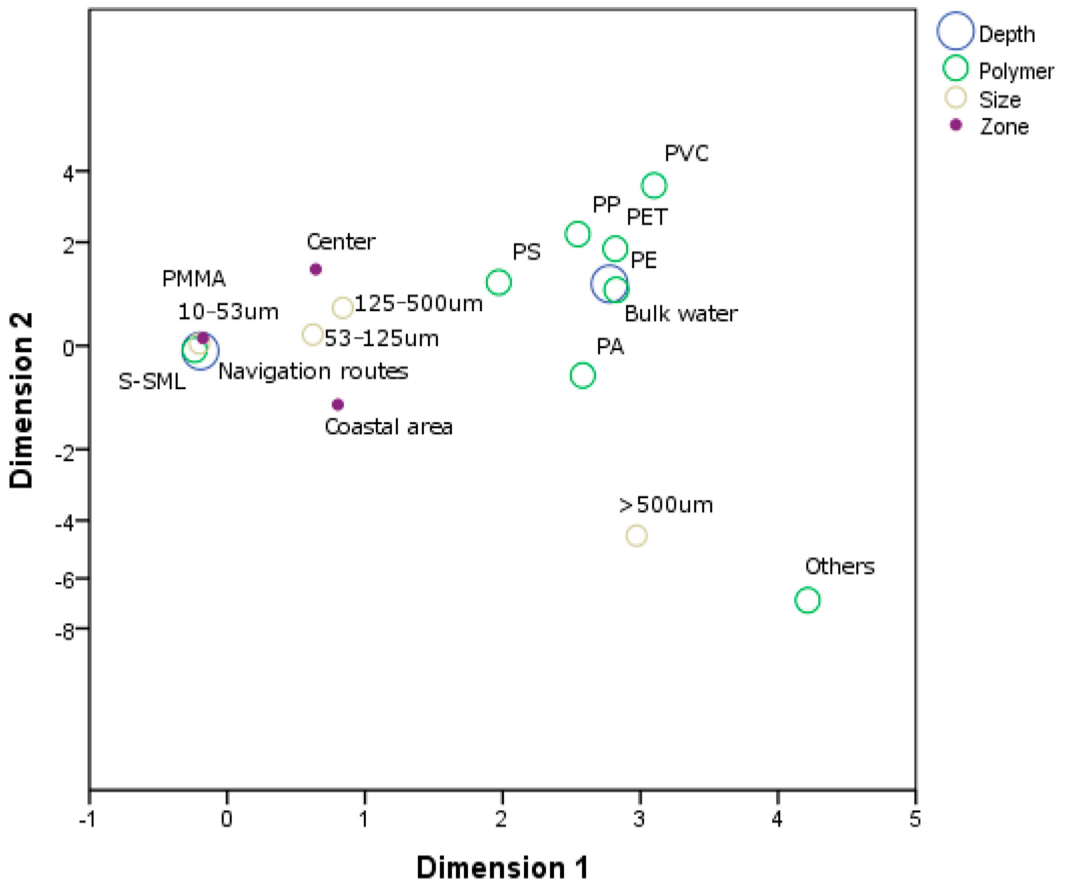

Figure S9 Correspondence analysis among depth (S-SML and bulk water), abundance of MPs of all kinds of polymer, size and sampling zones –

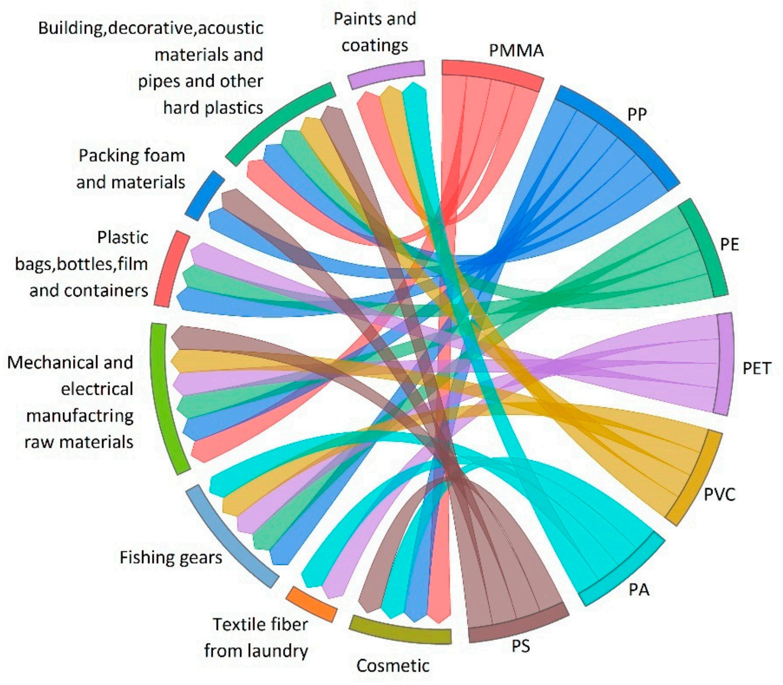

Figure S10 Source of MPs of all polymer type
